# Night-Time Monitoring System (eNightLog) for Elderly Wandering Behavior

**DOI:** 10.3390/s21030704

**Published:** 2021-01-20

**Authors:** James Chung-Wai Cheung, Eric Wing-Cheong Tam, Alex Hing-Yin Mak, Tim Tin-Chun Chan, Will Po-Yan Lai, Yong-Ping Zheng

**Affiliations:** 1 Department of Biomedical Engineering, Faculty of Engineering, The Hong Kong Polytechnic University, Hong Kong 999077, China; yongping.zheng@polyu.edu.hk; 2 Jockey Club Smart Ageing Hub, Department of Biomedical Engineering, The Hong Kong Polytechnic University, Hong Kong 999077, China; eric.tam@polyu.edu.hk (E.W.-C.T.); alexmak.mak@polyu.edu.hk (A.H.-Y.M.); tctimchan@polyu.edu.hk (T.T.-C.C.); will.lai@polyu.edu.hk (W.P.-Y.L.)

**Keywords:** elderly, fall, dementia, wandering, night monitoring, bed exiting, nursing home, ultrawideband radar, remote sensing

## Abstract

Wandering is a common behavioral disorder in the community-dwelling elderly. More than two-thirds of caregivers believe that wandering would cause falls. While physical restraint is a common measure to address wandering, it could trigger challenging behavior in approximately 80% of the elderly with dementia. This study aims to develop a virtual restraint using a night monitoring system (eNightLog) to provide a safe environment for the elderly and mitigate the caregiver burden. The eNightLog system consisted of remote sensors, including a near infra-red 3D time-of-flight sensor and ultrawideband sensors. An alarm system was controlled by customized software and algorithm based on the respiration rate and body posture of the elderly. The performance of the eNightLog system was evaluated in both single and double bed settings by comparing to that of a pressure mat and an infrared fence system, under simulated bed-exiting scenarios. The accuracy and precision for the three systems were 99.0%, 98.8%, 85.9% and 99.2%, 97.8%, 78.6%, respectively. With higher accuracy, precision, and a lower false alarm rate, eNightLog demonstrated its potential as an alternative to physical restraint to remedy the workload of the caregivers and the psychological impact of the elderly.

## 1. Introduction

Wandering is a common behavioral disorder that imposes hazards to older adults, particularly with dementia. Wandering means aimless ambulation in a living facility and is one of the most common symptoms of dementia. Wandering can also occur in people who needed assistive devices or wheelchairs [1]. The prevalence of wandering was 17.4% in community-residing seniors [2], 50% in those with severe dementia [3], and 63% in community dwellers [4]. It was reported that 70% of the caregivers regarded wandering as a risk for the care of the patients [5].

Wandering is one of the most exhausting behavioral symptoms for caregivers [6]. Although researchers attempted to investigate wandering from biomedical, psychosocial, and person–environment interaction perspectives, the etiology of wandering remains poorly understood. Some literature explained wandering as a dysfunction of spatial perception and memory [1]. Circadian rhythm disturbances, particularly sleep, have also been investigated as a basis for wandering [7]. From a psychosocial viewpoint, internal discomforts such as the need for toileting assistance or the need to search for a familiar place or person, especially when coupled with external factors such as noise, could drive the patient into wandering conditions [8]. The risk of falls in wandering individuals is higher than that of the non-wanderers [9], while the risk of fracture was also doubled in wanderers [10].

Falling is the major contributor to morbidity and mortality induced by wandering, although dementia could be an independent factor. Studies found that 28% of people aged over 65 years fall each year [11], while the number increased to 42% for those aged over 75 years [12]. The average rate of falls in the elderly with dementia in nursing homes was reported as 4.05 falls per year, compared to 2.33 falls per year for the elderly without dementia [13,14]. Older people with dementia also have a lower recovery rate after a fall in comparison with those without dementia [15]. Dementia-associated wandering and falls induce enormous costs in clinical care and elderly service. Limiting elderly people’s ambulation (restraint) is a widely accepted measure to compromise the short handedness of supporting staff, which contradicts the promotion of physical activities to combat the reduction in physical functioning [16].

Restraint is one of the most commonly used measures to address the issues with wandering and falls. Physical restraint is defined as any device adjacent to or attached to a person’s body that cannot be easily removed by the person and is deliberately designed to restrict the person’s freedom of movement and/or prevent the person from accessing their body normally [17]. Common types of physical restraint used in hospital and nursing home settings include bilateral bedside rails, trunk restraint, chair-boards composed of a chair with a fixed tray table [18], boxing gloves, and straitjackets [19]. In Hong Kong, physical restraint was applied to about 20% of the residents in homes for the elderly, to minimize the risk of elderly wandering and thus falls [20].

Increasing ethical concerns and negative impacts have been reported on the use of restraint. Up to 80% of the elderly with dementia developed challenging behaviors with physical restraint [21]. People in restraints reported negative feelings and psychological trauma such as helplessness, depression, fear, anger, anxiety, demoralization, and the loss of dignity [22]. Adverse reactions such as screaming, fighting, and extreme agitation could be triggered that endangers the person and his/her caregivers [23].

The minimization of physical restraints was enforced by the Joint Commission and the Food and Drug Administration (FDA) of the United States [24]. For instance, straitjacket were less allowed to be used for mentally disabled people [25]. Side rails were also discouraged since people can go over or be entrapped by the side rails, causing severe injury [26,27]. A new strategy of keeping the patient’s bed in a lower position could supplement side rails [28].

In summary, we believe that the emergence of technology can support wanderers’ safety and avoid the negative impact of physical restraint by using a virtual restraint. The overall objective of this study is to develop a noncontact, non-physical restraining night monitoring system (eNightLog system) that can alert the medical staff of potential danger of wandering and wandering-induced falls. This could support caregivers and provide a safe environment for the elderly, although it may not directly prevent falls from happening. The system’s innovation lies in its ability to identify whether an elderly person is within the dedicated zones by an algorithm using integrated images and signals from different sensors to detect potential wandering. Although bed-exiting events may not necessarily represent wandering, they also require attention from the caregivers. The system can generate different levels of alert to facilitate the prevention of wandering and related falls. The rationale for dedicating nighttime is because of the higher chance of abnormal nighttime behavior and a higher caregiver burden [29,30]. The sensitivity, specificity, accuracy, and precision of eNightLog were evaluated and compared to the existing detection system in a controlled laboratory setting.

## 2. Materials and Methods

### 2.1. Development of Night Time Monitoring System (eNightLog)

The eNightlog system was developed using remote sensing approaches for circumventing any physical contact, making it considerably less demanding for setup and less suspicion of being labeled and monitored. The system consisted of two sensors and a computer unit as main hardware with supporting software and algorithm. The sensors used include an infra-red 3D time-of-flight (ToF) sensor (Kinect V2 sensor, Microsoft, Redmond, WA, USA, with its RGB camera in sensor thoroughly concealed), and an Impulse-Radio Ultra-Wideband (UWB-IR) sensor (Xethru, Novelda, Norway). The UWB-IR sensor was designed to monitor the respiration rate and sleeping quality and support detection of the subject’s movement and presence. We collected the respiration rate and sleeping quality data from the UWB-IR sensor which serve as the presence value in the algorithm. The software was coded with Microsoft Visual C# (Microsoft, Redmond, WA, USA), and was designed to support image processing, connectivity, posture and position classification, alarm, and system configuration.

**Figure 1:**
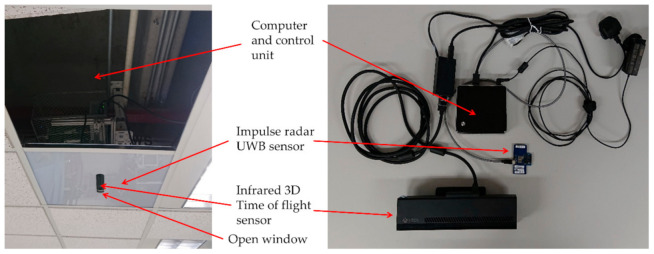
The eNightLog system setup concealed in the suspended ceiling with a windowed plastic tile.

The system hardware was hidden in the suspended ceiling with a narrow window to allow emission and receiving of infrared light (Figure 1). As impulse radar signals can pass through the non-metallic obstacle without a decline in signal quality, no opening was created in the ceiling tile for it. Privacy and position were the prime factors to be contemplated in monitoring. The images captured by the 3D ToF sensor were a depth map, consisting of a two-dimensional array with a width of 512 pixels and a height of 424 pixels as the size, and the depth values as contents.

If there were occlusions in the line of sight, the information of the overlapping objects in the rear position would be lost since a depth image was acquired from a single viewpoint, thus a depth image is normally referred to as 2.5D instead of 3D. In this application, since the system was ceiling-mounted, the chance of overlapping objects, particularly multiple subjects, dwindled substantially. The multiple reflection paths which may cause inaccuracy in the depth map were dramatically curtailed as well. The X, Y pixels, and depth value were recast to length, width, and height, respectively.

**Figure 2:**
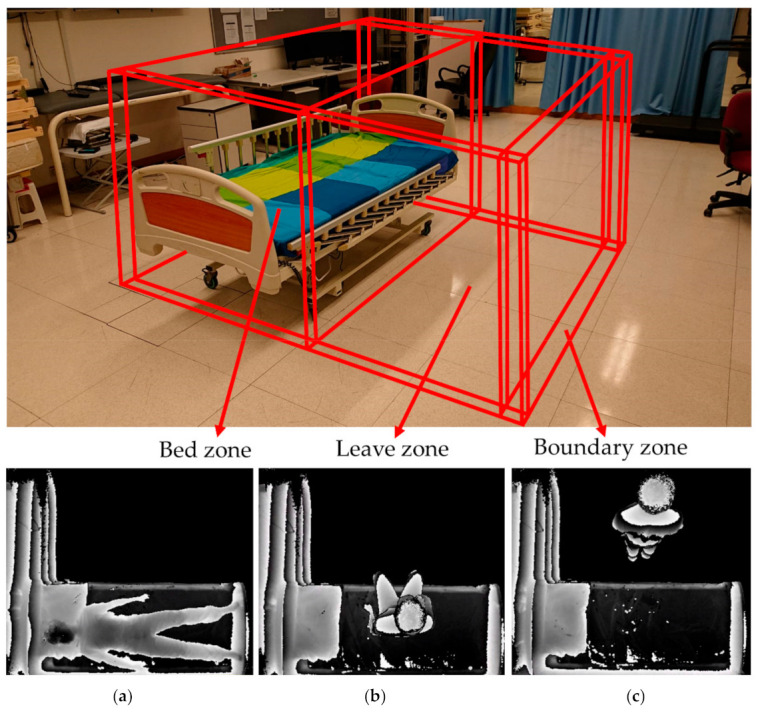
Illustration of three monitor zones, including the bed zone, leave zone, and boundary zone created by eNightLog system. (Bottom) Three key postures captured by the eNightLog system including (**a**) sleeping posture; (**b**) sitting posture; (**c**) standing posture.

The depth information was converted into greyscale by normalizing the range from the closest detected object to the farthest detected object. The 3D ToF sensor data were then revamped into a solid silhouette with contour shading to protect patients’ privacy, making the images uninterpretable for identification. The system segmented three sets of virtual boundaries, including the bed zone, leave zone, and boundary zone that acted as virtual fences for monitoring (Figure 2). In double-bed settings, the zones would not overlap.

### 2.2. Algorithm of Night Time Monitoring System (eNightLog)

**Figure 3:**
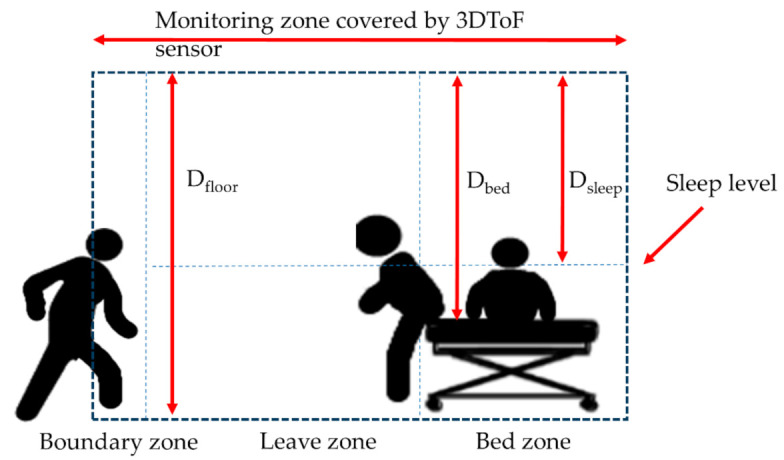
Main parameters used in eNightLog monitoring zone.

The algorithm used the decision tree approach with information from depth map and boundaries for classifying different positions and status. The status included sleeping, lying, sitting, standing, exiting, and others. There were different levels of alarm. As shown in Figure 3, a yellow alarm indicated that the subjects were located between the bed zone and the leaving zone which represented that the subjects sat on the edge of the bed or stood by the bed and may be going to exit the bed. A red alarm indicated the subjects departed the leave zone and crossed the boundary zone. The subjects totally leaving the zones could represent wandering or other potential risks. In this way, the activities near the bed (leave zone) would only give rise to the yellow (first level) alarm. Some statuses were used as internal statuses and thus not displayed.

The primary software functions of the eNightLog were streamed data processing and state classification. The definitions of parameters used in Algorithms A1 and A2 are shown in Table 1. Figure 3 shows the fundamental parameters including D_floor_, D_bed_, and D_sleep_ used in the algorithms. Other parameters in Table 1 were devised according to the fundamental parameters and corresponding zone area. Algorithm A1 (Appendix A) utilized depth streaming data to calculate different parameters for determining the different states and formations of the greyscale image. To calculate the parameters of P_bed zone_, P_leave zone_, and P_boundary_, we shall find out D_offset_ and D_floor_ first by the Kinect sensor. A background reference image B was captured and subtracted to each new image to remove background stationary objects. At the initial stage, five seconds of streaming data from both UWB-IR and 3D ToF sensor data were acquired to determine whether any motion occurred by objects being moved or the presence of the subject in the period, to ensure generating a background mask. Then, the first frame captured by 3D ToF in the period would be stored as the background. The parameter F’ was determined by the current frame subtracted by the stationary background. The parameters including P_depth_, P_above sleep_, P_leave zone_, and P_boundary zone_ can then be calculated by the pixels in the corresponding extents and continuously be updated for each new frame acquired. These parameters were then passed to Algorithm A2 (Appendix A) to determine the state. These parameters were subsequently stored and would be used to compare with the parameters calculated in the next frame. With reference to the thresholds of sleep level and boundary, the system can determine the current position and status of subject. A corresponding alarm is sequentially issued.

In this study, only exiting and other status were involved in the study design and performance evaluation with a conventional bed-exiting detection device, including pressure mat and infrared fence. An alert with a corresponding timestamp was issued and sent when a person was exiting or entering the bedside zone. We expected that the alert will be generated promptly because the system was a lightweight algorithm architecture. Two computers were running software locally for recording, display status, and alarming. The status and alarm data were sent in the form of network message attachment with status and timestamp to corresponding servers for data storage using Network-attached storage (NAS) and visualization in a local computer network in this validation study.

The silhouette formatted video frame rate was set at six frames per second for the two laboratory studies. The experimenter may mislabel the status due to the complexity of the scenarios and the lengthy process. Therefore, videos were recorded using a camcorder in laboratory experiment studies to provide redundancy ground truth. Data analysis panel software was developed for data visualization and manual confirmation of the status by studying both the videos and the status stored together.

### 2.3. Control (Physical Bed Exiting Detection System)

**Figure 4:**
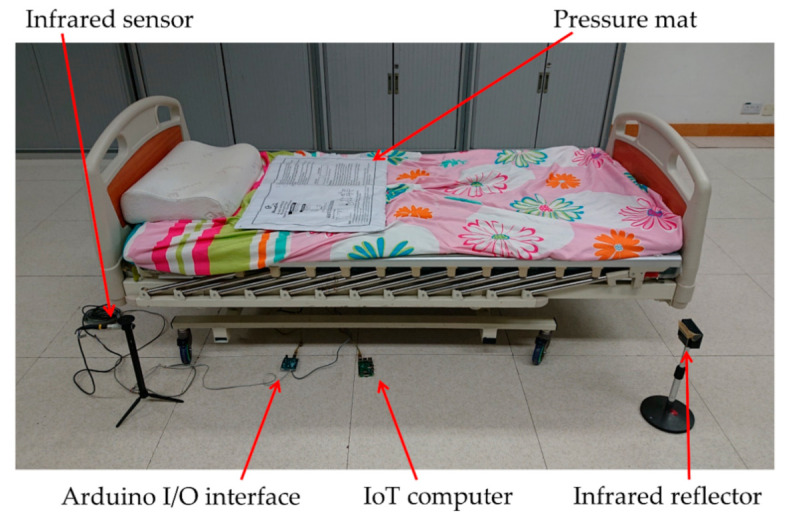
The hardware configuration of the conventional bed exiting detection system.

As shown in Figure 4, a conventional bed exiting detection system (CBD), infrared fence, and pressure mat was employed to compare the performance with eNightLog in the single-bed settings. The conventional system comprised of pressure mat (WarmCare, Pressure Pad for Contin Model WCP-B365-NC20, China) and infrared sensor (RS Pro Retro-reflective Photoelectric Sensor 4m Detection range NPN & PNP = NC\No IP67 Barrel Style, Radionics Ltd., Dublin, Ireland). The sensors were connected to Arduino board (Arduino Corp., Somerville, MA, USA) for reading status change while generating a timestamp, and linked to a Raspberry Pi single board IoT computer (Raspberry Pi Foundation, Cambridge, UK), which acted as the server for eNightLog and the conventional bed exiting detection system to record the status and time stamp for repository. A specific sequence of signal changes from both pressure mat and infrared sensor must be observed to determine whether the subjects were getting off the bed. Both the pressure mat and the infrared sensor would display an on-status whenever the subject sat on the bed side. The pressure mat would display an off-status as the subjects stood up, and subsequently, the infrared sensor would display an off-status as subjects exited the monitored zone.

A camcorder (DCR-SR42, Hard Disk Drive Handycam Camcorder, Sony, Tokyo, Japan) was used to record all activities and the screen of the monitor for analysis. This would help classify the status when the serial status change was occurring within a very short period of time in the conventional bed exiting system.

### 2.4. Evaluation of eNightLog System

#### 2.4.1. Participants

The experiments consisted of a single-bed setting and double-bed setting tests. For the single-bed setting test, a total of 30 healthy young subjects were recruited for the test, including 17 males. The average age for males was 23.5 ± 2.4 years while that of females was 23.0 ± 2.6 years. The average height was 175.2 ± 4.5 cm for males and 157.2 ± 4.1 cm for females.

For the double-bed setting test, there were 30 more healthy participants recruited in the experiment which played an interferer role in the experiment. The average age of these subjects is 23.4 ± 8.4 years for males (13), and 21.6 ± 5.7 years for females (17). The average height was 172.3 ± 5.7 cm for males and 157.3 ± 5.3 cm for females.

All subjects reported no physical disabilities or chronic disease. All subjects were recruited consecutively in the authors’ institution. The human subject experiment protocol for this study was reviewed and approved by The Human Subjects Ethics Sub-committee from The Hong Kong Polytechnic University (No. HSEARS20171019005). The written and informed consent were obtained from all participants.

#### 2.4.2. System Setup and Bed Setting

**Figure 5:**
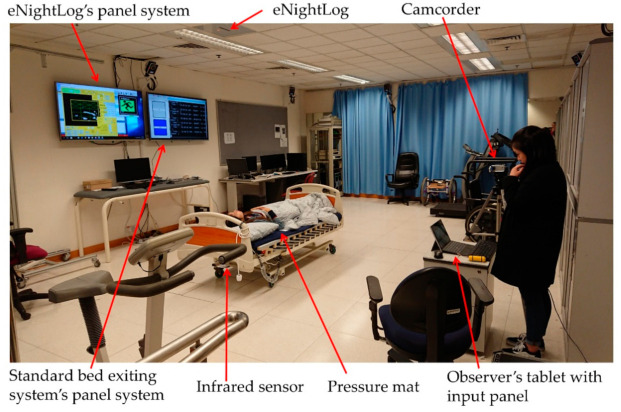
Setup of the experiment for eNightLog evaluation.

For the single-bed setting, the evaluation and the setup of the eNightLog system and the control were conducted simultaneously. An electric rehabilitation bed (length = 196 cm, width = 90 cm, height of bed surface = 55 cm) was positioned under the monitor zone of the eNightLog system. The sensors of eNightLog system was placed 270 cm above ground level (Figure 5). On the other hand, the pressure mat was placed on the bed where the subject was lying. The position of the pressure mat was checked and adjusted to the initial position in each trial. The infrared sensor was set up on the left side of the bed, where the subject could only enter or exit the bed in that direction. Two monitors were installed to display the status and changes corresponding to the eNightLog system and the conventional bed exiting detection system. In addition, a camcorder was set to record the subject’s activities and the monitor screens. An observer was to instruct the subject to perform activities predefined in a protocol. The observer also recorded the time when bed-exiting occurred by pressing the control button on a tablet.

**Figure 6:**
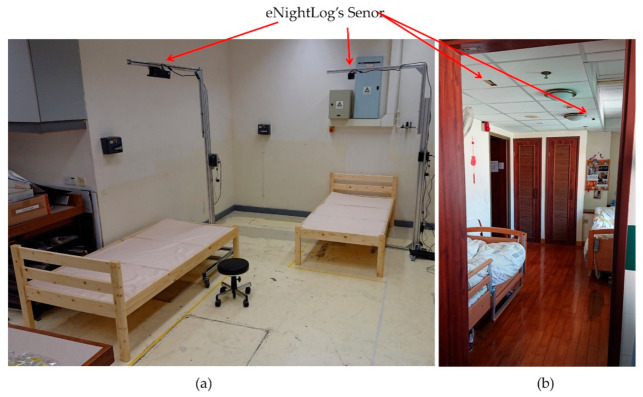
(**a**) Simulated double-bed setting with tapes on the ground to denote the walls and entries; (**b**) The actual double-bed room in the hostel served as the setting in this experiment.

In the double-bed setting, only the two eNightLog systems were installed on each bed to access its ability to overcome interference from other subjects or visitors. The CBD was not intervened in the double-bed setting. The bed size and the bed placement were arranged in diagonal corners (as shown in Figure 6) according to that of a private hostel for the elderly with dementia to simulate a realistic setting. The other two corners were mimicked as the toilet entry and the room entry, respectively. If the subjects were exiting, they must pass through the different monitoring zones of the eNightLog systems (Figure 6).

**Figure 7:**
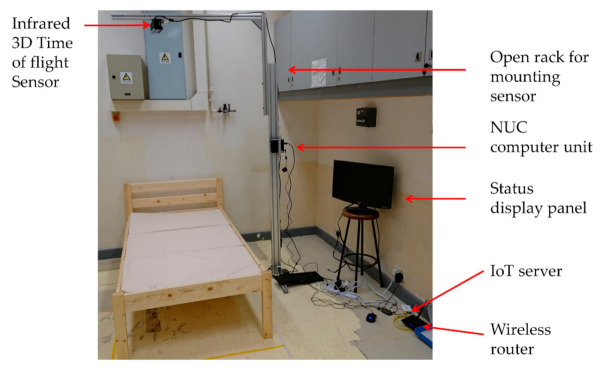
Simulated double bed experiment equipment setup.

The two wooden beds with dimensions of length 196 cm, width 90 cm, and height 38 cm were set under the full monitoring bed zones of the two eNightLog systems. The sensors of eNightLog systems were installed 230 cm above ground level supported by two aluminum racks, which simulated the suspended ceiling position for the elderly hostel to install the system. Two eNightLog systems were wirelessly connected to the IoT server responsible for managing and forwarding system messages. The software would display the subjects’ status monitored by the two eNightLog systems (Figure 7). Additionally, the camcorder was set to record activities within the room as the ground truth for data checking. An observer instructed the subjects to perform activities formulated by a modified protocol from the single-bed setting detailed in the next section. Silhouette formatted videos generated from two eNightLog systems were regarded as another reference.

#### 2.4.3. Procedures and Protocols

For the single-bed setting, we simulated nine bed-time activity scenarios according to experience and information given by the hostel’s caregivers. The background of each scenario and details of the activities involved in each scenario are shown in Table 2 and Table 3.

Each subject was required to read and remember the steps of each scenario. Subjects were asked to perform each scenario three times. The observer would remind the subjects if necessary. Each scenario was randomly picked to form a unique sequence of scenarios for each subject by the observer. A total of 27 trials (9 scenarios × 3 times) were performed by each subject. Since Sc3 consisted of two bed-exiting moves, the total number of activities for evaluation was 900 (9 + 1 scenario moves × 3 times × 30 subjects).

For the double-bed setting, we modified three simulated bed-time activity scenarios (Sc3, Sc7, and Sc8) to fit the double-bed room setting as shown in Table 4. Those scenarios were designed to simulate the interference of other individuals including subjects of the other bed or caregivers. The systems would only identify the targeted subject while the other individuals were recognized as visitors. A pair of subjects were randomly and independently assigned to the double-bed scenarios. However, the combination of scenarios among the two subjects would not be repeated. Therefore, there was a total of 45 combinations of scenarios (i.e., a summation series towards 9). Since Sc3 had one more additional bed-exiting move and it appeared ten times in the combination, the total number of moves for each pair was 300 (3 trials × 45 combinations × 2 paired-subject + 3 trials × 10 additional moves by Sc3). Therefore, the total number of moves was 4500 (300 × 15 pairs).

#### 2.4.4. Evaluation

The systems recorded all bed-exiting activities and marked the timestamps. A confirmed event of bed-exiting activities was asserted by the observer. Only one marking would be made on each scenario move even though multiple false alarms on a single scenario move could be possible. A true activity means the actual activity was detected correctly by the system; whereas, a false activity means activity detected by the system without a corresponding actual event occurred. The detection metrics are defined as follows:

- True Positive (TP): The system correctly detected a true bed-exiting move.
- False Positive (FP): The system incorrectly detected a false bed-exiting move.
- True Negative (TN): The system correctly recognized a non-bed-exiting move.
- False Negative (FN): The system incorrectly recognized a non-bed-exiting move.

To evaluate the performance of both systems on bed-exiting detection, the accuracy, precision, sensitivity, and specificity were calculated by the Equations (1)–(4).

(1) $$Sensitivity\;=\;\frac{TP}{TP+FN}\;\times \;100\%$$

(2) $$Specificity\;=\;\frac{TN}{TN+FP}\;\times \;100\%$$

(3) $$Accuracy\;=\;\frac{TP+TN}{TP\;+TN\;+\;FP\;+\;FN}\;\times \;100\%$$

(4) $$Precision\;=\;\frac{TP}{TP+FP}\;\times \;100\%$$

Sensitivity is the percentage of all actual activities correctly detected. Specificity is the percentage of correctly not detected activities when no activity occurred. Accuracy measures how good of the ability differentiation the correctly detected and not detected the activity. Precision indicates the ability to correctly detected activity, which is an actual activity.

## 3. Results

During the two laboratory experiments, all frames were analyzed along with the video captured as ground truth. In the single- and double-bed experiments, there were altogether 900 and 4500 bed-exiting performed, respectively. Table 5 shows a detailed overview of the two experiment results and presents the sensitivity, specification, accuracy, and precision for eNightLog and the control.

The accuracy of eNightLog of the bed-exiting event in the experiments was higher than 98.8% in both settings, indicating excellent accuracy. The accuracy of the double-bed study was slightly lower than that of the single-bed study.

On the other hand, the accuracy of eNightLog in both settings was still remarkably higher than that of CBD-coupled bed-exiting detection system. In addition, eNightLog outperformed CBD in terms of specificity and precision.

## 4. Discussion

Controlling false alarms is actually of paramount importance in caring. The high accuracy, high precision, and low false alarm rate in this study showed the potential of the eNightLog in clinical and home applications. Exiting study suggested that the low specificity of the pressure sensor or the CBD triggered a lot of false alarms [31]. This would lead to alarm fatigue that hindered long-term fall prevention. Pressure sensors also required constant maintenance, and repositioning [31], thus greatly increasing caregivers’ workload. In addition, audible alarms could cause distress to residents, especially those with cognitive impairment [32]. In terms of sensitivity and false negatives, the CBD system and eNightLog performed similarly well in the single-bed study. Both systems achieved high performance in correctly recognizing bed-exiting event. This is particularly important for preventing wandering and injury. In hostels, beds are normally aligned with the head close to the wall and even distributed space between beds with ample space. Full bed-length fences are installed on the side of the bed to prevent the elderly from falling out and colliding with other residents. However, in land-scarce cities like Hong Kong, irregular alignment in beds is common, particularly for private hostels. The two-bed setting in this study was imitating a real hostel’s two-bed room. The two beds were not aligned on the same side but parked in two opposite corners to maximize the separation. Elderly people on the inner bed would pass through the bed zone of the other bed that is likely trigger a false alarm of the infrared fence. The number of false alarms in the CBD system, therefore, is expected to be much higher.

There was a 2.8-fold increase (from 7/1800 to 100/9000) in the number of false alarms of the eNightLog system from the single-bed to the double-bed setting. Apparently, visitor and neighbor activities considerably interfered with system recognition that explained why the double-bed setting had slightly lower accuracy, precision, and specificity. In a real hostel, multiple beds in the same room are even more common. Previous studies showed that the use of cameras in elderly people’s living environment raised privacy concerns [33]. We believed that detecting the positions on the bed and bed lifts was sufficient to trace whether subjects were within the dedicated area and compromised with the privacy issue. In additions, we introduced a top view video using raw data from 3D time of flight sensor to produce an image in form of solid silhouette with contour shading (Figure 2c) and coalesce into a video. Although a silhouette image might enable identification, body features, facial and clothing features were masked and could not be used for personal identification. This processed video could pave the way for wandering detection with sufficient privacy in the clinical experiment in the future study.

It shall be noted that our current system focused on the presence of the elderly in the zones to identify bed-exiting activities and produce appropriate alarms. Although our research was dedicated to elderly people wandering, any bed-exiting activities should also require attention. Current detection strategies focused on a subject who stands, sits, lies, in a position in an ideal location of the 3D time flight sensor’s field of vision, while the real conditions vary and are more dynamic. For instance, caregivers or the elderly could use wheelchairs and walking aids. These items could be occasionally left inside the monitor zone, misleading the system to recognizing a human’s presence. Line of slight could also be temporarily blocked by movable furniture. The system may also track caregivers or movable items instead of the targeted subject. Wearable devices for caregiver could be used for the system to facilitate better identification. The array of 3D time-of-flight sensors could also mitigate this problem. It is, however, more challenging to implement.

UWB-IR sensor in eNightLog was used for detecting small vibrations such as breathing, which was also exploited for supporting elderly presence detection and improved the detection. This could reduce the problem of obstacles. Some studies attempted to integrate pressure sensors and depth sensors to identify a subject’s posture and body build [34]. Among different sensors used to detect human presence, activities, and vital signs, UWB-IR can produce these measurements through walls and furniture [35]. It also does not have a problem with privacy or removal of wearable vital sign-monitoring devices by the wearer. It can detect posture changes and could possibly for detection bed-exiting [36].

Table 6 shows the performance comparison among the different systems used in previous studies. Among these studies, eNightLog outperformed all others in accuracy and precision compared to realistic simulated scenarios. Sensitivity was almost the same as the CBD system in this study. Intriguingly, eNightLog had similar specificity compared to existing systems using a pressure mat, infrared fence, and multiple pressure mat, and thermal array with the ultrasonic sensor. However, existing studies did not simulate different types of bed-exiting events. The CDB in this study has significantly lower specificity than the infrared fence and multiple pressure mats system because of the visitor-checking and subject-rolling-over scenarios. Although pressure sensors and infrared are good alternatives without tight physical constraints, the effectiveness of infrared bed-exiting sensors and pressure mat for alerting bed-exiting was of particular concerned. The U.S. Food and Drug Administration (FDA) has received plenty of reports on false and incorrect alarms [37]. Infrared bed exiting sensors and pressure mats can be integrated to improve sensitivity that is as yet unsuccessful [37]. In addition, elderly can easily spot the devices, giving them a strong negative feeling of being monitored.

Telecare products, including sensors and location devices, which help manage wander walking are increasingly common facilities in hostels. Position-change alarm products, including chair and bed sensor pads, bedside alarm mats, alarms clipped to a resident’s clothing, seatbelt alarms, and infrared bed-exiting alarm, are essential to alerting caregivers in cases of bed-exiting. In fact, these devices are now acting as a form of restraint. It helps the caregiver restrict people to exit certain areas by alerting them to stop the elderly in time which may also be referred to as virtual restraint. The virtual restraint devices are mostly audible to the residents in the hostel. It might have unintended consequences of inhibiting freedom of movement. The residents may be afraid to set off the alarm that could be a nuisance to other residents or staff, restricting their physical motion. Negative potential outcomes may still occur such as loss of dignity, decreased mobility, bowel and bladder incontinence, sleep disturbances, confusion, fear, agitation, anxiety, or irritation in response to the sound of the alarm as a warning for escape [20], leading to an ethical issue on restrictions of freedom of the elderly with dementia.

eNightLog was designed to avoid using any form of informative alarm to be recognized by the elderly. An alarm or warning was sent to the nurse station or caregivers’ mobile device without creating unnecessary alarm sound to all residents. It can also provide a vibration alarm to the caregivers through the mobile device. Auto-playing of voice-based alarms will be introduced after training sessions for the hostel where the field study is planned to be conducted. eNightLog can further improve its performance with the wearable device and deep learning technique. A simple wearable device composed of accelerators can provide posture information and recognize fall events [42]. The system can verify posture and moving speed from the wearable device, which can be attached or embedded into clothing. In fact, the wearable device can provide a range of functions and measurements, including reminders of scheduled tasks, measurement of core and skin body temperature, heart rate, respiration rate, blood pressure, and urine volume. This information is crucial for caregivers and physicians to offer better care. eNightLog’s image can potentially provide a range of possible further development such as fall detection in the bedside zone, a fall out of a bed, sleeping quality study (including frequency of rolling over on the bed), and periodic movement from leg [43]. Since the virtual fence zone of eNightLog is adjustable, minor changes to bed position and height can be easily accommodated without hardware reinstallation. eNightLog can also be linked with a lighting and door access system to further support the elderly who wake up at night. Recently, the convolution neural network has been studied to predict bed exiting according to in-bed behavior [44]. With the deep learning technique, the false alarm rate could be further reduced. Indeed, the deep learning technique can further provide information on various in-bed behaviours such as sleeping disorders. In addition, the caregiver activities and performance could be possibly derived, such as time to respond to the event.

The 3D ToF sensor mounted at height 2.3 m and 2.7 m were investigated. The eNightLog system sensor must be set above 2.2 m to ensure coverage of the full body and periphery for collecting sufficient data to determine subject status and posture. Sensors with a wider angle of view could be used to replace the current sensor in monitoring double deck bed or hanging bed. Alternatively, the algorithm could be modified so that the system could be installed onto the wall. It will give more flexibility for installation at home. In Hong Kong, most of the hostel residents are not arranged with a single room because of the cost and property size. It is not uncommon for four or more residents to share the same room. Night wandering of roommates and hostel staff members’ regular visits could unintentionally trigger the alarm. False alarm rate could be minimized by aligning the bed to limit ways from getting off the bed. However, with a highly dense living environment and hostels converted from buildings for other purposes, non-aligned settings were common. Therefore, further study on different bed position combinations should be conducted.

On the other hand, it is also possible to monitor two subjects with a single 3D ToF sensor by installing the system well-above 3m. However, it involved completely rewriting the current algorithm, which currently cannot track multiple subjects lying on the bed(s) with a single sensor. The silhouette image or video clip can be replaced by an avatar for enhancing protection of privacy. The image and video clip can be substituted with the detailed status sign to further preserve privacy. A longitudinal study in multiple centers will be conducted to evaluate the system performance further. The images collated could be further used to develop the deep learning algorithm to improve the accuracy in future field study and identify more status or behavior that requires monitor.

## 5. Conclusions

The ageing population already draws great attention because of the tremendous impact on economics and social burden, particularly with many elderly people living with dementia and consequences of fall injuries. Physical and chemical restraints are the most common measures to tackle these problems. However, ethical issues and effectiveness are growing concerns. It is very demanded to prevent someone from wandering or independently bed-exiting without physical restraint or chemical restraint. However, it is still a necessary measure for preventing patient’s night activities and fall. Virtual restraint is a good alternative. Existing systems have various limitations and thus cannot be widely and reliably used.

The eNightLog system was successfully developed with high sensitivity and specificity to tackle the aforementioned restraint approaches’ problems. It was designed to support monitoring at night in the nursing home, which currently suffer from the ageing of caregivers, lack of sufficient night-shift staff, and a dramatic increase in demand for dementia service. It also supports the night-shift staff members to monitor all elderly people with a round-the-clock check on elderly status, preventing night wandering and eliminating regular setup of bed-exiting monitor devices. It also minimized infringement on the privacy of monitored elderly people by using silhouette images. It does not require mounting of any sensors on the body or clothes of the elderly people monitored. Our studies found that it outperformed existing products with similar purposes or other devices in similar studies. It has potential for other applications such as in sleeping disorder monitoring and being connected to other environmental control devices as a new element of the smart home. Elderly health monitoring is equally important to behaviour monitoring. Some health issues could relate to sleep disturbance or behaviour [45]. The system can be enhanced by integration with different health monitoring, such as snoring [46] and coughing monitoring [47]. We assumed that elderly and young people performed similar movement during bed exiting in this laboratory-based investigation despite that the young subjects could have generally higher speed and level of activity. In fact, we are currently expanding our work regarding elderly people to optimize the system. Further development using different sensors and learning algorithms may extend its potential to other applications. Regardless of the physical and mental condition of the elderly, they deserve a safe and comfortable sleeping environment. eNightLog system is a step towards this goal.

## 6. Patents

The eNightLog has filed a Chinese application invention patent and has been published (No.201710706823.X).

## Figures and Tables

**Table 1:** List of notations and definitions used in the algorithm of eNightLog system.

Notation	Definition
F	Extract current frame from Kinect depth streaming
B	Background frame from Kinect depth streaming without human presence
F’	Frame subtracted from B to remove the stationary background
D_floor_	Distance between floor and 3D ToF sensor
D_bed_	Distance between the bed and 3D ToF sensor
D_sleep_	Distance between sleep level and 3D ToF sensor
D_offset_	Constant value in cm determined by measuring in cm of a person height when lying on the bed with 2 cm margin added
P_bed zone_	Number of depth point above sleep level (D_sleep_)
P_leave zone_	Number of depth point within leave zone
P_boundary zone_	Number of depth point in boundary
P_depth_	Number of depth point of current frame = P_bed zone_ + P_leave zone_ + P_boundary zone_
P^’^_depth_	Number of depth point of next frame
M_above sleep level_	Motion above sleep level detected
M_leave zone_	Motion in leave zone detected
M_boundary zone_	Motion in boundary detected
pTh_motion_	Number of change of depth point in the detect zone
B_bpm_	Breath per minute measured by ultra-wideband impulse radar
S_state_	Current state: ‘sleep locked’, ‘sit’, ‘existing bed’, ‘leave’, ‘others’, ‘both leave’ ‘sleep locked’ denotes bed exiting monitoring started. ‘sit’ denotes the subject sit on the bed. ‘exiting bed’ denotes the subject existing bed. ‘leave’ denotes subject left monitor zones. ‘others’ denotes non-monitoring subject (caregiver) entered the monitor zones. ‘both leave’ denotes non-monitoring subject (caregiver), and the subject left monitor zones.
S^’^_state_	Previous state
pTh_above sleep level_	The threshold of depth point above sleep for distinguishing lying and sitting
pTh_leave zone_	The threshold of depth point in leave zone for determining subject entering leave zone
pTh_boundary_	The threshold of depth point in the boundary for determining subject moving in/out boundary zone

**Table 2:** Simulated bedtime activity scenario.

Scenario	Purpose of Simulation
Sc1	Simple scenario—Simulates the process of walking in, sleeping, waking up and exiting bed without any interruption or physical activities.
Sc2	General scenario—Based on the first scenario with the addition of rolling over on bed, which is the most common activity during sleep.
Sc3	Urination scenario—Simulates when the sleeping process was interrupted by the need to go to toilet. Subject was required to leave the monitored zone and return to bed. It leaded to generating bed-exiting record twice.
Sc4	Drinking scenario—Stimulates when the sleeping process was interrupted by picking up a glass of water to drink and return to sleep without exiting the bed.
Sc5	Use of device scenario—Simulates when the sleeping process was interrupted by picking up a remote control or other device to switch on/off electrical appliance and return to sleep without exiting the bed.
Sc6	Blockage of nasal cavity scenario—Simulates when the sleeping process was interrupted by waking up to sneeze, clean up and return to sleep without exiting the bed.
Sc7	Caregiver helping scenario—Simulates when a caregiver supported walking and transferring to bed, and later visited to check on the subject. At the end, subject woke up and left the bed without caregiver’s assistance.
Sc8	Caregiver checking scenario—Simulates when the sleeping process was interrupted by a caregiver visiting, checking, and exiting. At the end, subject woke up and left the bed without caregiver’s assistance.
Sc9	Cough scenario—Simulates the sleeping process interrupted by waking up for coughing, drinking from a glass of water and return to sleep without exiting the bed.

Sc denotes scenario.

**Table 3:** Nine different scenarios in the experiment. Row number denotes the step in each scenario to be followed.

	Sc1	Sc2	Sc3	Sc4	Sc5	Sc6	Sc7	Sc8	Sc9
1	Walk in	Walk in	Walk in	Walk in	Walk in	Walk in	Walk in with caregiver	Walk in	Walk in
2	Sit on bed	Sit on bed	Sit on bed	Sit on bed	Sit on bed	Sit on bed	Sit on bed	Sit on bed	Sit on bed
3	Move to centre	Move to centre	Move to centre	Move to centre	Move to centre	Move to centre	Move to centre	Move to centre	Move to centre
4	Lying	Lying	Lying	Lying	Lying	Lying	Lying	Lying	Lying
5	Cover with quilt	Cover with quilt	Cover with quilt	Cover with quilt	Cover with quilt	Cover with quilt	Cover with quilt	Cover with quilt	Cover with quilt
6	Sleep	Sleep	Sleep	Sleep	Sleep	Sleep	Caregiver check	Sleep	Sleep
7	Wake up	Roll over	Roll over	Roll over	Roll over	Roll over	Caregiver leave	Visitor walk in check	Roll over
8	Flip over quilt	Wake up	Wake up	Wake up	Wake up	Difficult breathing	Sleep	Visitor leave	Itchy throat
9	Sit on bed	Flip over quilt	Flip over quilt	Flip over quilt	Flip over quilt	Wake up	Visitor walk in check	Sleep	Wake up
10	Move to edge	Sit on bed	Sit on bed	Sit on bed	Sit/lay on bed	Sit on bed	Visitor leave	Wake up	Cough
11	Stand up	Move to edge	Move to edge	Drink water	Use of device	Sneeze/cough	Sleep	Flip over quilt	Drink water
12	Leave bed	Stand up	Stand up	Sleep	Sleep	Sleep	Wake up	Sit on bed	Sleep
13		Leave bed	Leave bed	Roll over	Roll over	Roll over	Flip over quilt	Move to edge	Roll over
14			Repeat step 1 to 13	Wake up	Wake up	Wake up	Sit on bed	Stand up	Wake up
15				Sit on bed	Sit on bed	Sit on bed	Move to edge	Leave bed	Sit on bed
16				Move to edge	Move to edge	Move to edge	Stand up		Move to edge
17				Stand up	Stand up	Stand up	Leave bed		Stand up
18				Leave bed	Leave bed	Leave bed			Leave bed

Sc denotes scenario.

**Table 4:** Modified stimulated bed-time activity scenario.

Scenario	Purpose of Simulation
Sc3	Urination scenario—Simulates when the sleeping process was interrupted by the need to go to toilet. Subject was required to leave the monitored zone, enter and exit the other monitored zone, and then return to bed. It leaded to generating bed-exiting record twice.
Sc7	Caregiver helping scenario—Simulates when a caregiver supported walking and transferring to bed, and later visited to check on both subjects before returning to the door location. At the end, subject woke up and left the bed without caregiver’s assistance.
Sc8	Caregiver checking scenario—Simulates when the sleeping process was interrupted by a caregiver visiting, checking and exiting. The caregiver was required to check on both subjects before returning to door location. At the end, subjects woke up and left the bed without caregiver’s assistance.

Sc denotes scenario.

**Table 5:** Bed exiting event recognition results and evaluation results.

Setting and Outcome	Single Bed Study	Single Bed Study	Double Bed Study
Device	CBD	eNightLog	eNightLog
Total number of events detected	1800	1800	9000
True Positive (TP)	890	889	4495
True Negative (TN)	657	893	4400
False Positive (FP)	243	7	100
False Negative (FN)	10	11	5
Accuracy	85.9%	99.0%	98.8%
Precision	78.6%	99.2%	97.8%
Sensitivity	98.9%	98.8%	99.9%
Specificity	73.0%	99.2%	97.8%

CBD denotes conventional bed exiting detection system.

**Table 6:** Comparison with different studies for posture and bedside event recognition.

Sensor(s)	Source	Accuracy	Precision	Sensitivity	Specificity
Infrared fence	[38]	N/A	N/A	85.3%	96.2%
Pressure mat	[38]	N/A	N/A	90.4%	99.3%
Pressure sensor	[39]	N/A	N/A	96%	95.5%
CBD	This study	85.9%	78.6%	98.9%	73.0%
Infrared fence and multiple pressure mats	[38]	N/A	N/A	92.3%	99.4%
Thermal array and ultrasonic sensor	[40]	95.5%	93.8%	71.4%	99.3%
RFID	[31]	N/A	N/A	93.8%	90.8%
Kinect	[41] #	98.8%	N/A	N/A	N/A
eNightLog	This study *	99.0%	99.2%	98.8%	99.2%

# Patient get up from the bed event; * Single bed laboratory setting. CBD—conventional bed exiting detection system.

## Data Availability

The data presented in this study are available on request from the corresponding author. The data are not publicly available due to privacy of subjects.

## Appendix A

The appendix is the pseudo codes of the algorithms.

**Algorithm A1** Streamed data processing
1. Input: F, D_floor_, D_offset,_ D_bed_
2. Output: P_bed zone_, P_leave zone_, P_boundary_
3. F = Extract Kinect depth streamed data
4. Convert F to grey scale image
5. Determine D_floor_ and D_bed_ by reading Kinect sensor
6. D_sleep_ = D_bed_ − D_offset_
7. Repeat // Setup loop
8. B = Extract one frame from Kinect depth streaming to background buffer
9. Until no motion in 5 seconds period time
10. Repeat // Main loop
11. B_bpm_ = UWB-IR sensor reading
12. Resultant frame F^’^ = F − B
13. P_depth_ = count the pixels with value
14. P_bed zone_ = count the pixels with value above D_sleep_ in bed region from F’
15. P_leave zone_ = count the pixels with value above D_floor_ in leave zone from F’
16. P_boundary_ = count the pixels with value above D_floor_ in boundary from F’
17. P_depth_ = P_above sleep_ + P_leave zone_ + P_boundary zone_
18. Call (Algorithm A2)
19. P^’^_depth_ = P_depth_
20. P’_bed zone_ = P_bed zone_
21. P’_leave zone_ = P_leave zone_
22. P’_boundary_ = P_boundary_
23. Until break

**Algorithm A2** State determination
1. Input: P_bed zone_, P_leave zone_, P_boundary,_ B_bpm,_ P_depth_, P^’^_depth,_ pTh_motion_, S^’^_state_
2. Output: S_state_
3. If ((P_depth_ − P^’^_depth_ ≤ pTh_motion_) or S^’^_state_ = ‘others’) and P_boundary_ = 0 and P_bed zone_ = 0 and P_leave zone_ = 0 and B_bpm_ > 0
4. S_state_ = ‘sleep locked’
5. Else
6. If P_bed zone_ > pTh_above sleep level_ and P_leave zone_ < pTH_leave zone_
7. S_state_ = ‘sit’
8. break
9. If P_leave zone_ > pTH_leave zone_ and P_bed zone_ < pTh_above sit level_
10. S_state_ = ‘exiting bed’
11. break
12. If S^’^_state_ = ‘exiting bed’ and P_leave zone_ = 0 and P_bed zone_ = 0
13. S_state_ = ‘leave’
14. break
15. If Not (S^’^_state_ = ‘leave’) and P_boundary_ > pTh_boundary_
16. S_state_ = ‘others’
17. break
18. If S^’^_state_ = ‘others’ and P_leave zone_ = 0 and P_bed zone_ = 0 and P_boundary_ = 0 and B_bpm_ = 0 S_state_ = ‘both leave’ Break
19. endif
20. S’_state_ = S_State\_
21. Display status

## References

[1] Cipriani G., Lucetti C., Nuti A., Danti S. (2014). Wandering and dementia. Psychogeriatrics.

[2] Klein D.A., Steinberg M., Galik E., Steele C., Sheppard J.M., Warren A., Rosenblatt A., Lyketsos C.G. (1999). Wandering behaviour in community-residing persons with dementia. Int. J. Geriatr. Psychiatry.

[3] Teri L., Larson E.B., Reifler B.V. (1988). Behavioral disturbance in dementia of the Alzheimer’s type. J. Am. Geriatr Soc..

[4] Hope T., Tilling K.M., Gedling K., Keene J.M., Cooper S.D., Fairburn C.G. (1994). The structure of wandering in dementia. Int. J. Geriatr. Psychiatry.

[5] Utton D. (2009). The design of housing for people with dementia. J. Care Serv. Manag..

[6] Rolland Y., Gillette-Guyonnet S., Nourhashemi F., Andrieu S., Cantet C., Payoux P., Ousset P.J., Vellas B. (2003). Wandering and Alzheimer’s type disease. Descriptive study. REAL.FR research program on Alzheimer’s disease and management. Rev. Med. Interne.

[7] Tetewsky S.J., Duffy C.J. (1999). Visual loss and getting lost in Alzheimer’s disease. Neurology.

[8] Phillips V.L., Diwan S. (2003). The incremental effect of dementia-related problem behaviors on the time to nursing home placement in poor, frail, demented older people. J. Am. Geriatr. Soc..

[9] Colombo M., Vitali S., Cairati M., Perelli-Cippo R., Bessi O., Gioia P., Guaita A. (2001). Wanderers: Features, findings, issues. Arch. Gerontol. Geriatr..

[10] Wick J.Y., Zanni G.R. (2006). Aimless excursions: Wandering in the elderly. Consult. Pharm..

[11] Prudham D., Evans J.G. (1981). Factors associated with falls in the elderly: A community study. Age Ageing.

[12] Downton J.H., Andrews K. (1991). Prevalence, characteristics and factors associated with falls among the elderly living at home. Aging.

[13] Morris J.C., Rubin E.H., Morris E.J., Mandel S.A. (1987). Senile dementia of the Alzheimer’s type: An important risk factor for serious falls. J. Gerontol..

[14] van Doorn C., Gruber-Baldini A.L., Zimmerman S., Hebel J.R., Port C.L., Baumgarten M., Quinn C.C., Taler G., May C., Magaziner J. (2003). Dementia as a risk factor for falls and fall injuries among nursing home residents. J. Am. Geriatr. Soc..

[15] Shaw F.E. (2002). Falls in cognitive impairment and dementia. Clin. Geriatr. Med..

[16] Dionyssiotis Y. (2012). Analyzing the problem of falls among older people. Int. J. Gen. Med..

[17] Kwok T., Bai X., Chui M.Y., Lai C.K., Ho D.W., Ho F.K., Woo J. (2012). Effect of physical restraint reduction on older patients’ hospital length of stay. J. Am. Med. Dir. Assoc..

[18] Kwok T., Mok F., Chien W.T., Tam E. (2006). Does access to bed-chair pressure sensors reduce physical restraint use in the rehabilitative care setting?. J. Clin. Nurs..

[19] Yan E., Kwok T., Lee D., Tang C. (2009). The prevalence and correlates of the use of restraint and force on hospitalised older people. J. Nurs. Healthc. Chronic. Illn..

[20] Feng Z., Hirdes J.P., Smith T.F., Finne-Soveri H., Chi I., Du Pasquier J.N., Gilgen R., Ikegami N., Mor V. (2009). Use of physical restraints and antipsychotic medications in nursing homes: A cross-national study. Int. J. Geriatr. Psychiatry.

[21] Barnes T.R., Banerjee S., Collins N., Treloar A., McIntyre S.M., Paton C. (2012). Antipsychotics in dementia: Prevalence and quality of antipsychotic drug prescribing in UK mental health services. Br. J. Psychiatry.

[22] Lancaster G.A., Whittington R., Lane S., Riley D., Meehan C. (2008). Does the position of restraint of disturbed psychiatric patients have any association with staff and patient injuries?. J. Psychiatr. Ment. Health Nurs..

[23] Andrews G.J. (2006). Managing challenging behaviour in dementia. BMJ.

[24] Dimant J. (2003). Avoiding physical restraints in long-term care facilities. J. Am. Med. Dir. Assoc..

[25] Foderaro L.W. Hospitals Seek an Alternative to Straitjacket. http://www.nytimes.com/1994/08/01/nyregion/hospitals-seek-an-alternative-to-straitjacket.html?pagewanted=all.

[26] US Food & Drug Adminstration Recommendations for Consumers and Caregivers about Bed Rails. https://www.fda.gov/medical-devices/bed-rail-safety/recommendations-consumers-and-caregivers-about-bed-rails.

[27] Talerico K.A., Capezuti E. (2001). Myths and facts about side rails. Am. J. Nurs..

[28] Tzeng H.M., Prakash A., Brehob M., Devecsery D.A., Anderson A., Yin C.Y. (2012). Keeping patient beds in a low position: An exploratory descriptive study to continuously monitor the height of patient beds in an adult acute surgical inpatient care setting. Contemp. Nurse.

[29] Neikrug A.B., Ancoli-Israel S. (2010). Sleep disorders in the older adult—A mini-review. Gerontology.

[30] Yaffe K., Falvey C.M., Hoang T. (2014). Connections between sleep and cognition in older adults. Lancet Neurol..

[31] Ranasinghe D.C., Shinmoto Torres R.L., Hill K., Visvanathan R. (2014). Low cost and batteryless sensor-enabled radio frequency identification tag based approaches to identify patient bed entry and exit posture transitions. Gait Posture.

[32] Shorr R.I., Chandler A.M., Mion L.C., Waters T.M., Liu M., Daniels M.J., Kessler L.A., Miller S.T. (2012). Effects of an intervention to increase bed alarm use to prevent falls in hospitalized patients: A cluster randomized trial. Ann. Intern. Med..

[33] Demiris G., Hensel B.K., Skubic M., Rantz M. (2008). Senior residents’ perceived need of and preferences for "smart home" sensor technologies. Int. J. Technol. Assess. Health Care.

[34] Wong D.W.-C., Wang Y., Lin J., Tan Q., Chen T.L.-W., Zhang M. (2019). Sleeping mattress determinants and evaluation: A biomechanical review and critique. PeerJ.

[35] Cho H.S., Park Y.J. (2018). Detection of Heart Rate through a Wall Using UWB Impulse Radar. J. Healthc. Eng..

[36] Baird Z. (2017). Human Activity and Posture Classification Using Single Noncontact Radar Sensor. Doctoral Dissertation.

[37] Capezuti E., Brush B.L., Lane S., Rabinowitz H.U., Secic M. (2009). Bed-exit alarm effectiveness. Arch. Gerontol. Geriatr..

[38] Lu C., Huang J., Lan Z., Wang Q. Bed exiting monitoring system with fall detection for the elderly living alone. Proceedings of the 2016 International Conference on Advanced Robotics and Mechatronics (ICARM).

[39] Hilbe J., Schulc E., Linder B., Them C. (2010). Development and alarm threshold evaluation of a side rail integrated sensor technology for the prevention of falls. Int. J. Med. Inf..

[40] Asbjorn D., Jim T. (2017). Recognizing Bedside Events Using Thermal and Ultrasonic Readings. Sensors.

[41] Ni B., Dat N.C., Moulin P. RGBD-camera based get-up event detection for hospital fall prevention. Proceedings of the 2012 IEEE International Conference on Acoustics, Speech and Signal Processing (ICASSP).

[42] Cheung C., Chan W.R., Chiu M., Law S., Lee T., Zheng Y. A three-month study of fall and physical activity levels of intellectual disability using a transfer belt-based motion recording sensor. Proceedings of the International Federation for Medical and Biological Engineering (IFMBE).

[43] Garn H., Kohn B., Dittrich K., Wiesmeyr C., Kloesch G., Stepansky R., Wimmer M., Ipsiroglu O., Grossegger D., Kemethofer M. 3D detection of periodic limb movements in sleep. Proceedings of the 2016 38th Annual International Conference of the IEEE Engineering in Medicine and Biology Society (EMBC).

[44] Chen T.X., Hsiao R.S., Kao C.H., Liao W., Lin D.B. Bed-exit prediction based on convolutional neural networks. Proceedings of the 2017 International Conference on Applied System Innovation (ICASI).

[45] Lee K.K., Birring S.S. (2010). Cough and sleep. Lung.

[46] Veauthier C., Ryczewski J., Mansow-Model S., Otte K., Kayser B., Glos M., Schöbel C., Paul F., Brandt A.U., Penzel T. (2019). Contactless recording of sleep apnea and periodic leg movements by nocturnal 3-D-video and subsequent visual perceptive computing. Sci. Rep..

[47] Thi T.H., Wang L., Ye N., Zhang J., Maurer-Stroh S., Cheng L. (2014). Recognizing flu-like symptoms from videos. BMC Bioinf..

